# Research on Temperature-Switched Dopamine Electrochemical Sensor Based on Thermosensitive Polymers and MWCNTs

**DOI:** 10.3390/polym15061465

**Published:** 2023-03-15

**Authors:** Haixiu Wang, Zufei Feng, Fupeng Lin, Yan Zhao, Yangfan Hu, Qian Yang, Yiming Zou, Yingjuan Zhao, Rong Yang

**Affiliations:** 1 School of Science, Xi’an University of Technology, Xi’an 710048, China; 2 International Research Center for Composite and Intelligent Manufacturing Technology, Institute of Chemical Power Sources, Materials and Engineering College, Xi’an University of Technology, Xi’an 710048, China

**Keywords:** PNIPAM, electrochemical sensor, dopamine

## Abstract

A temperature-controlled electrochemical sensor was constructed based on a composite membrane composed of temperature-sensitive polymer poly (N-isopropylacrylamide) (PNIPAM) and carboxylated multi-walled carbon nanotubes (MWCNTs-COOH). The sensor has good temperature sensitivity and reversibility in detecting Dopamine (DA). At low temperatures, the polymer is stretched to bury the electrically active sites of carbon nanocomposites. Dopamine cannot exchange electrons through the polymer, representing an “OFF” state. On the contrary, in a high-temperature environment, the polymer shrinks to expose electrically active sites and increases the background current. Dopamine can normally carry out redox reactions and generate response currents, indicating the “ON” state. In addition, the sensor has a wide detection range (from 0.5 μM to 150 μM) and low LOD (193 nM). This switch-type sensor provides new avenues for the application of thermosensitive polymers.

## 1. Introduction

Dopamine (DA) is an important neurotransmitter produced and released by the central nervous system which plays an important role in many physiological activities, such as emotions, learning, movement, behavior and memory [1,2]. Imbalances in human DA levels are closely related to many diseases and addictions, such as Parkinson’s disease [3], depression [4], senile dementia [5], and drug addiction [6]. Therefore, the rapid and accurate determination of dopamine concentration in vivo is of great significance. At present, there are many methods for the detection of dopamine, such as colorimetry [7], spectrophotometry [8], capillary electrophoresis [9], electrochemiluminescence [10], high-performance liquid chromatography [11], and fluorescence [12]. However, most analytical techniques have some shortcomings, such as high-performance liquid chromatography (HPLC), which requires large equipment and is expensive, and fluorescence analysis, which has a complex detection process and poor selectivity and effectiveness [12]. Based on the electrochemical activity of DA, the molecule contains easily oxidized phenolic hydroxyl, which can be detected by electrochemical analysis [13]. Electrochemical technology has many advantages, such as simplicity of operation, fast response to DA, low cost, high sensitivity, good stability, and availability of on-site monitoring [14]. However, the matrix contains some substances whose oxidation potential isclose to DA, such as uric acid and ascorbic acid, which may interfere with the detection of dopamine. When using electrochemical methods to detect dopamine, the glassy carbon electrode (GCE) needs to be modified.

Stimulus-responsive polymers refer to materials that produce specific responses to molecules or polymers as the external environment (temperature, light, humidity, magnetic field strength, pH, etc.) changes, thereby changing the chemical or physical properties of the materials themselves. They have received extensive attention [15,16,17,18] and have been widely used in many fields, such as drug transportation [19], bionic materials [20], and microfluidic devices [21]. The temperature-responsive polymer is the most widely used stimulus-responsive polymer with the clearest mechanism at present. Thermosensitive polymers are generally divided into two types: one where, when the temperature is lower than the critical temperature and the polymer is in a state of hydrophilic dissolution; when the temperature is higher than the critical temperature and the polymer is in a state of hydrophobic turbidity. At this point, the critical temperature is referred to as the Lower Critical Solution Temperature (LCST). The other where, polymer occurs phase transition near the critical temperature from low-temperature insoluble to high-temperature soluble, and the critical temperature at this point is called the Upper Critical Solution Temperature (UCST). When the temperature is changed near the polymer’s LCST or UCST, the hydrophilicity and hydrophobicity of the polymer will change. A temperature-reversible switching electrochemical sensor is prepared by combining the temperature-sensitive polymer with the electrochemical sensor. However, due to the poor conductivity of polymers they are usually combined with nanomaterials to form composite materials to improve the stability and sensing performance of sensors. The LCST of PNIPAM is 32 °C, which is close to the temperature of the human body. At the same time, PNIPAM is easy to prepare and cheap. It has good biocompatibility and is widely used [22]. For example, Dan prepared a temperature-switch electrochemical sensor based on a palladium–graphene composite and PNIPAM for the detection of 4-nitrophenol in water, showing better sensitivity, selectivity and long-term stability [23]. Mutharani used sonochemistry to prepare WO_2_@PS-co-PNIPAM for the temperature-controlled reversible “ON-OFF” electrochemical detection of β-Blocker metoprolol [24]. Carbon nanomaterials have excellent conductivity and electrochemical catalytic effects. When used as electrode materials, they can accelerate signal transduction through catalytic activity, conductivity and biocompatibility. Multi-walled carbon nanotubes (MWCNTs) are one-dimensional carbon nanomaterials with good conductivity, electrocatalytic activity and adsorption properties, which can accelerate signal transmission, improve sensing ability and improve sensor sensitivity.

In this paper, the temperature-sensitive polymer PNIPAM and carboxylated multi-walled carbon nanotubes (MWCNTs-COOH) were used to construct an intelligent electrochemical DA sensor with temperature control. Among them, multi-walled carbon nanotubes provide excellent electrochemical and electrocatalytic properties for sensors. The electrochemical process was successfully controlled by adjusting the temperature and the sensitive detection of DA was realized. More importantly, we achieved satisfactory results in drug analysis and the detection of actual human serum samples using this sensor.

## 2. Materials and Methods

### 2.1. Reagents and Apparatus

N-isopropylacrylamide (NIPAM), azodiisobutyronitrile (AIBN), n-hexane, acetone, cyclohexane and potassium chloride were purchased from Sinopharm Chemical Reagent Co., Ltd. (Shanghai, China). Carboxylated multi-walled carbon nanotubes (with an outer diameter of 20–30 nm and a length of 10–30 microns) were purchased from Chengdu Organic Chemical Co., Ltd. (Chengdu, China), Chinese Academy of Sciences. Disodium hydrogen phosphate and sodium dihydrogen phosphate were purchased from Tianjin Kemio Reagent Chemical Co., Ltd. (Tianjin, China). Potassium ferricyanide was purchased from Tianjin Kemio Reagent Chemical Co., Ltd. Dopamine hydrochloride was purchased from Shanghai Meiruier Chemical Reagent Co., Ltd. (Shanghai, China). The serum was obtained from Huada Forensic Identification Institute in Xixian New Area, Shaanxi Province.

We obtained CHI660D electrochemical workstation (Shanghai Chenhua Co., Ltd. (Shanghai, China)), DZ-2BC vacuum-drying oven (Tianjin Taist Instrument Co., Ltd. (Tianjin, China)), DF-101S heat-collecting constant-temperature heating magnetic stirrer (Shanghai Lichen Bangxi Instrument Science and Technology Co., Ltd. (Shanghai, China)), 85-2 digital display constant-temperature magnetic stirrer (Changzhou Yuexin Instrument Manufacturing Co., Ltd. (Changzhou, China)), and a ZEISS Gemini 300 SEM.

Three-electrode system was adopted for electrochemical tests, and glassy carbon electrode, platinum electrode and calomel electrode were purchased from Tianjin Aida Hengsheng Technology Development Co., Ltd. (Tianjin, China). The ultrapure water used in the experiment was prepared by Shuhuoquan’s YK-RO-B ultrapure water mechanism.

### 2.2. Preparation of PNIPAM

PNIPAM was obtained through free-radical polymerization: first, recrystallize the purchased NIPAM to remove the polymerization inhibitor, take an appropriate amount of NIPAM and add it into acetone/n-hexane (*v/v* = 1:1), wait for the solution to saturate, filter out the insoluble substances, seal it and put it in the refrigerator overnight, and the crystals obtained are NIPAM. Add cyclohexane (18 mL), recrystallized NIPAM (1.25 g) and AIBN (0.012 g) into the reaction flask, stir until completely dissolved, pass N_2_ for 30 min to remove oxygen, react for 2 h under N_2_ at 70 °C, stop the reaction, cool down, add acetone to dilute, drop the product into a large amount of n-hexane, generate white precipitate, repeatedly dissolve and precipitate three times, put it into a vacuum-drying oven at 60 °C and dry overnight, and the white product obtained is PNIPAM.

### 2.3. Preparation of Modified Electrode

First, polish the GCE with aluminum oxide powder (1 μm); then, clean ultrasonically in ethanol and H_2_O for 30 s and dry it at room temperature. Then, dissolve PNIPAM in ultrapure water (3 mg·mL^−1^) and ultrasonically treat the carboxylated multi-walled carbon nanotubes in ultrapure water for 1 h to obtain a uniform dispersion (2 mg·mL^−1^). Then, combine 10 μL carboxylated multi-walled carbon nanotubes dispersion and 10 μL PNIPAM solution, mix and disperse ultrasonically for 30 min. Then, drop the obtained dispersion onto the surface of the clean GCE. Finally, dry the prepared electrode at room temperature.

### 2.4. Main Test Methods

SEM characterization: Use a pipette gun to take the suspended liquid of the sample and drop it on the silicon wafer, dry it naturally in the room, spray gold, and stick the silicon wafer to the sample table with conductive adhesive for testing.

Electrochemical detection of DA: The traditional three-electrode system was used, in which the modified GCE was used as the working electrode, the platinum electrode as the auxiliary electrode, and the saturated calomel electrode as the reference electrode. The electrochemical determination of DA in 0.1 mol·L^−1^ PBS (pH 7.0) was carried out by cyclic voltammetry. The CV was carried out at a scan rate of 100 mV·s^−1^ in a scanning range of −0.2~0.6 V with 2 s standing time. On this basis, DPV was used to further detect DA with a potential range of 0–0.3 V and 2 s standing time. All water solutions were ultrapure water.

EIS analysis: The EIS measurements were carried out by applying the value of the open-circuit potential and recorded over a frequency range of 0.1 Hz to 10 kHz with an ac amplitude of 10 mV.

### 2.5. Analysis of Actual Samples

Take 200 μL serum and dilute to 20 mL with 0.1 M PBS (pH = 7). Test with DPV at 40 °C. In addition, a standard addition method was used to calculate the recovery rate.

## 3. Results

### 3.1. SEM Characterization

**Figure 1:**
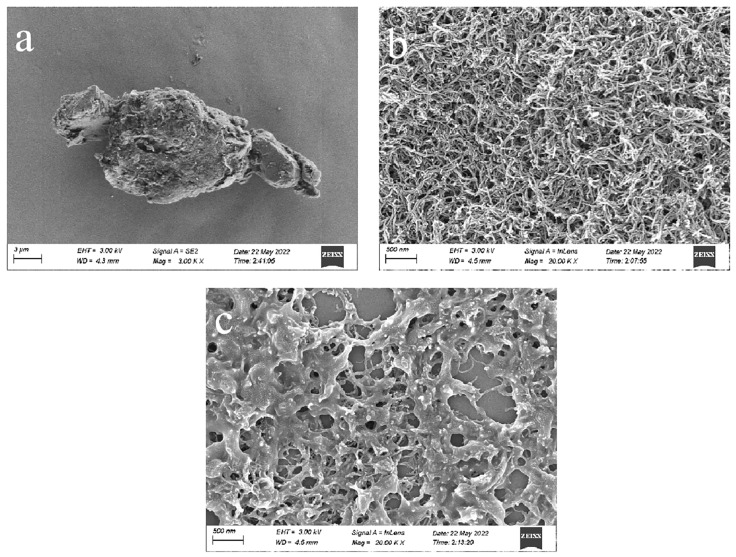
Scanning microscope characterization of different materials: (**a**) PNIPAM; (**b**) MWCNTs (COOH); (**c**) PNIPAM/MWCNTs (COOH).

As shown in Figure 1, the surface morphology of different materials was characterized by a scanning electron microscope (SEM). It can be seen that (a) the polymer PNIPAM is irregular in shape and rough in surface, (b) MWCNTs (COOH) is a uniform tubular structure, (c) PNIPAM in PNIPAM/MWCNTs is wrapped on the surface of MWCNTs (COOH) and the two are successfully compounded.

### 3.2. EIS Analysis

Electrochemical impedance spectroscopy (EIS) is a crucial electrochemical-measurement technology. It is used to describe changes in the electron-transfer rate at the interface of different modified electrodes and can reflect the conductivity of modified electrode materials. The EIS spectrum is generally composed of semicircles and straight lines: the diameter of the semicircles in the high-frequency area represent the electron-transfer resistance; the straight lines in the low-frequency area represent diffusion control.

**Figure 2:**
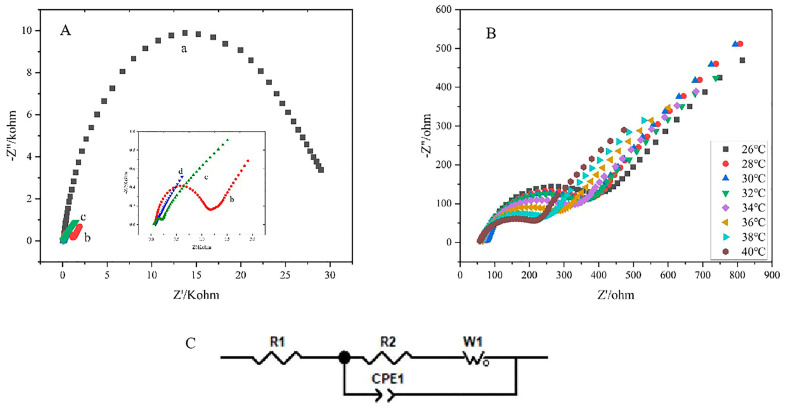
(**A**) EIS spectra observed in 5 mM [Fe(CN)_6_]^3-/4-^ redox probe containing 0.5 M KCl at 40 °C. (a) PNIPAM/GCE; (b) GCE; (c) PNIPAM/MWCNTs(COOH)/GCE; (d) MWCNTs(COOH)/GCE. (**B**) EIS spectra of PNIPAM/MWCNTs (COOH)/GCE in 5 mM [Fe(CN)_6_]^3−/4−^ redox probe containing 0.5 M KCl at various temperatures (26−40 °C). (**C**) equivalent electrical circuit.

As shown in Figure 2, the EIS spectrum of different materials was investigated, and Fe (CN)_6_^3−/4−^ acted as the electroactive probe containing 0.5 M KCl at the frequency range from 0.1 Hz to 10 kHz. The EIS of the GCE, the MWCNTs (COOH)/GCE, PNIPAM/GCE, and PNIPAM/MWCNTs (COOH)/GCE were tested (Figure 2A). In order to explore the change in R value, we used a software to fit the curve (the equivalent circuit diagram is shown in Figure 2C). In the equivalent circuit, R_2_ is the charge–transfer resistance; R_1_ is the electrolyte resistance; Cdl is a double-layer capacitor; and W_1_ is Warburg impedance. Overall, PNIPAM/GCE has the largest impedance value—its impedance value is 28,010 ohm through fitting—because PNIPAM is a macromolecular polymer with poor conductivity, which greatly hinders the electron transfer at the electrode interface and reduces the electron-transfer rate, so the impedance value is greater than that of the GCE. On the contrary, carboxylated carbon nanotubes have good conductivity and promote the electron-transfer rate on the electrode surface, so the impedance of MWCNTs (COOH)/GCE is very small and its impedance value is close to 0. The impedance value of PNIPAM/MWCNTs(COOH)/GCE (110.7 ohm) is between that of the GCE (1010 ohm) and the MWCNTs (COOH). This shows that, compared with PNIPAM/GCE, PNIPAM/MWCNTs(COOH)/GCE introduced the MWCNTs(COOH), which improved the conductivity of the composite membrane and reduced the obstruction in the electron transmission. This also shows that the temperature-sensitive polymer with poor conductivity and the MWCNTs(COOH) with excellent conductivity have a very good synergy, which was proof that the two successfully formed composite materials with a good electrochemical performance.

Figure 2Bshows the EIS of the composite modified electrode at different temperatures. It is obvious that with the increase in temperature, the semicircle radius decreases and the impedance value decreases. When the temperature remains lower than 32 °C—that is, lower than the LCST of PNIPAM—the impedance value does not decrease significantly when the temperature is raised; however, when the temperature is higher than 32 °C, the hydrogen bond between PNIPAM and the water molecules is broken, the polymer segment shrinks, reducing the distance between the MWCNTs(COOH) and the electrode surface, the electron-transfer rate increases, and the impedance value decreases.

### 3.3. Dopamine-Detection Behavior of Different Materials

**Figure 3:**
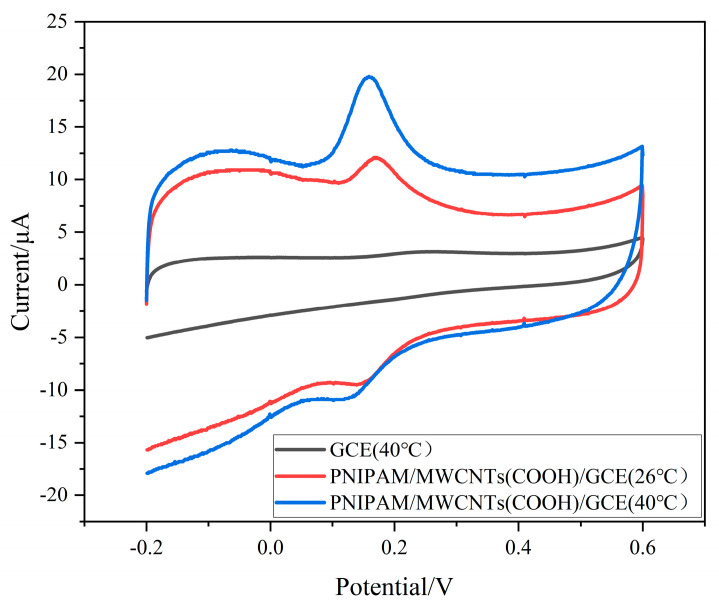
Electrochemical response of GCE and PNIPAM/MWCNTs (COOH)/GCE to DA; electrolyte solution: 0.1 M PBS (pH = 7.0); scan rate: 0.1 V/s; DA concentration: 30 μM.

As shown in Figure 3, the CV of DA in the GCE and PNIPAM/MWCNTs (COOH)/GCE showed that both of them had an electrochemical response to DA, but there was a pair of redox peaks on the composite modified electrode, while the GCE only had a weak oxidation peak, which showed that the composite modified electrode had good electrocatalytic activity for DA and could improve the electron-transfer rate on the electrode surface. However, although the redox response of PNIPAM/MWCNTs (COOH)/GCE occurred at 26 °C and 40 °C, the peak value of PNIPAM/MWCNTs (COOH)/GCE’s oxidation peak was higher at 40 °C and the peak value was also more obvious. The composite modified electrode was temperature-sensitive, which was consistent with the above AC impedance test.

### 3.4. Effect of Scan Rate and pH

**Figure 4:**
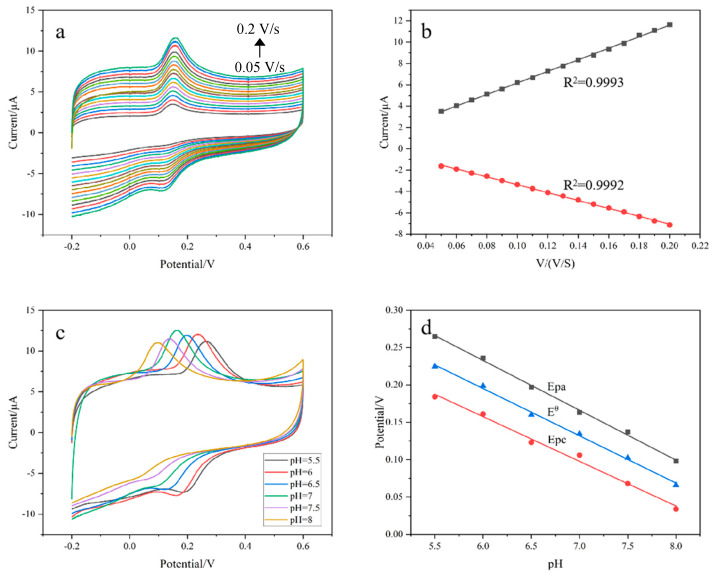
(**a**) The CV voltammograms of DA at different scan rates on the composite modified electrode, with rates from 0.05 V/s to 0.2 V/s—electrolyte solution: 0.1 M PBS (pH = 7.0), DA concentration: 30 μM, Solution temperature: 40 °C; (**b**) the linear relationship between oxidation-peak and reduction-peak currents and scan rate; (**c**) the CV voltammograms of DA at different pHs on the composite modified electrode—DA concentration: 30 μM, scan rate: 0.1 V/S, solution temperature: 40 °C; (**d**) the linear relationship between potential and pH.

Figure 4a explores the cyclic voltammetry of DA on the composite modified electrode at different scan rates. It can be seen that in the range of 0.05–0.20 V/s, the electrochemical response gradually increases with the increase in scan rate. Further analysis shows that the oxidation-peak current and the reduction-peak current have a good linear relationship with the scan rate (Figure 4b). The relationship is as follows:

Ipa = 54.143V (mV/s) + 0.7154 (R^2^ = 0.9993)

Ipc = −36.946V (mV/s) + 0.3263 (R^2^ = 0.9992)

This shows that the redox process of DA on the composite electrode is controlled by adsorption.

Figure 4c shows the CV voltammograms of DA on the composite modified electrode at different pHs. It can be seen that the redox peak potential of DA shifts negatively with increases in solution pH, indicating that protons participate in the redox reaction of DA. By exploring the relationship between pH values and peak-potential values, we can find the oxidation-peak potential value Epa, the reduction-peak potential Epc and the formula potential E^θ^. The linear equation with pH values is:

Epa = 0.63241 − 0.06663 pH (R^2^ = 0.9976)

Epc = 0.51612 − 0.05977 pH (R^2^ = 0.9918)

E^θ^ = 0.57417 − 0.0632 pH (R^2^ = 0.9973)

**Figure 5:**
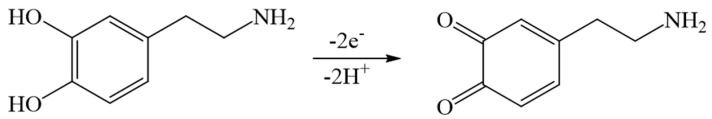
The feasible electrochemical−oxidation mechanism of DA.

The slopes of Epa, Epc and E^θ^ are −66.63, −59.77 and −63.2 mV/pH, respectively. According to the relationship between pH values and peak potential [25], the number of protons and electrons involved in the redox reaction of DA are the same. This proves that the redox reaction of DA on the surface of the composite material is an “isoelectronic-isoproton” process. In accordance with the literature [1], the feasible electrochemical-reduction mechanism of DA is shown in Figure 5. Since the oxidation-peak current at pH = 7 is the largest and it is close to the pH of the human body, we chose pH = 7 as the optimal condition.

### 3.5. Temperature Response of DA on Composite Modified Electrode

**Figure 6:**
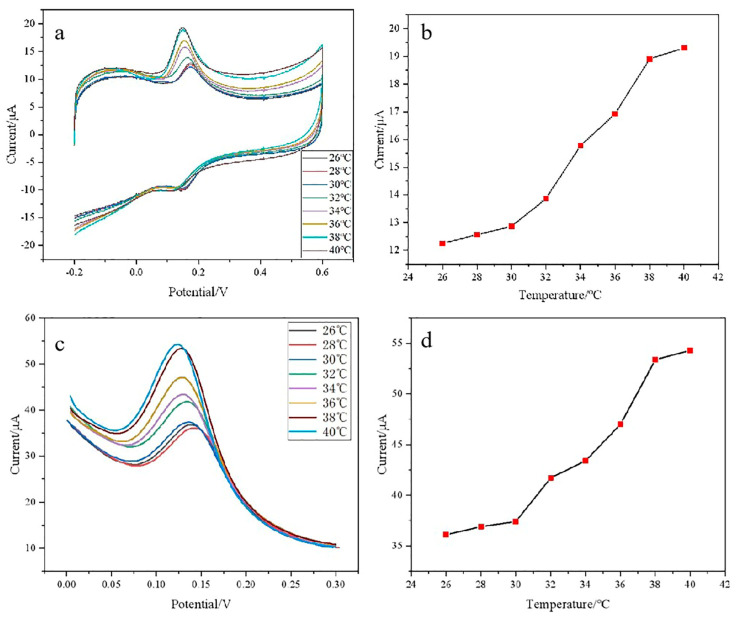
(**a**) CV voltammograms, (**c**) DPV voltammograms of the composite modified electrode to DA at different temperatures, and (**b**,**d**) the broken line diagrams of their respective peak currents and corresponding solution temperatures—DA concentration: 30 μM.; scan rate: 0.1 V/S; electrolyte solution: 0.1 mol·L^−1^ PBS (pH = 7.0).

In order to investigate the temperature-response characteristics of the composite modified electrode, the electrochemical response of DA on PNIPAM/MWCNTs (COOH)/GCE at different temperatures was studied by cyclic voltammetry. It can be seen from Figure 6a,b that PNIPAM/MWCNTs (COOH)/GCE has good temperature-response characteristics. When the solution temperature is 32 °C and below, the oxidation peak is small. With the increase in temperature, the oxidation-peak current also increases. This is because when the temperature is lower than 32 °C, PNIPAM and water molecules form hydrogen bonds and the chain segment extends, which increases the thickness of the composite film, thus hindering electron transfer. With the increase in temperature, the hydrogen bonds break and the chain segment of PNIPAM shrinks. With the decrease in the thickness of the composite film, the electron-transfer rate and oxidation-peak current increase. This shows that the detection of dopamine by PNIPAM/MWCNTs (COOH)/GCE has the properties of a temperature-responsive zipper switch. Furthermore, DPV with a higher sensitivity was used to study the temperature-switching effect of DA. It can be seen from Figure 6c,d that when the temperature is lower than 32 °C the peak current is small, indicating that the electrode surface is in the “OFF” state; when the temperature is higher than 32 °C, the redox peak current is significantly increased, the electron-transfer rate is accelerated, and the electrode surface presents an “ON” state, which is consistent with the results obtained by cyclic voltammetry. The above phenomena explain how temperature can control the electrochemical reaction state of DA on the composite modified electrode.

### 3.6. Temperature-Reversible Switch Response of DA on Composite Modified Electrode

**Figure 7:**
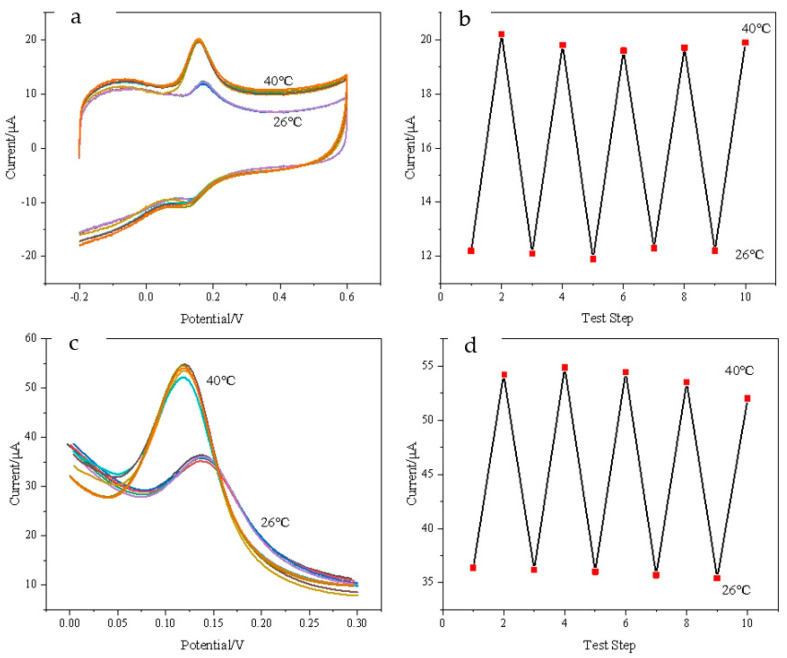
(**a**) CV voltammograms, (**c**) DPV voltammograms, and (**b**,**d**) the oxidation−peak currents of DA at 26 °C and 40 °C for ten cycles—DA concentration: 30 μM; scan rate: 0.1 V/s; electrolyte solution: 0.1 mol·L^−1^ PBS (pH = 7.0).

Figure 7 shows that the composite modified electrode has a reversible “ON-OFF” characteristic controlled by temperature. The switching reversibility of DA is studied at 26 °C and 40 °C by CV (Figure 7a) and DPV (Figure 7c), respectively. It can be seen from Figure 7b,d that the peak current is small at 26 °C and the electrochemical reaction is in the “OFF” state; when the temperature is at 40 °C, the peak current increases significantly and the detection of DA is in the “ON” state. When the solution temperature is raised and lowered repeatedly, the electrochemical detection of DA shows that the CV and DPV response signals of DA are not significantly attenuated at high temperatures, which indicates that the modified electrode has a reversible temperature-switching response.

**Figure 8:**
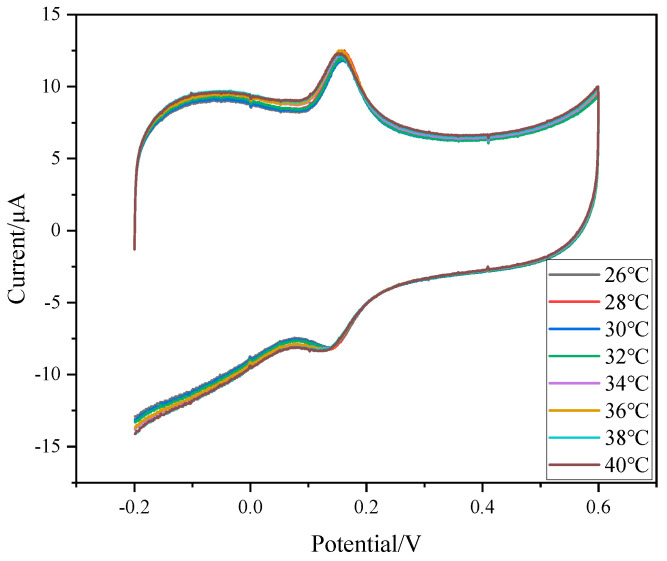
CVs of MWCNTs (COOH)/GCE to DA at different temperatures—DA concentration: 30 μM; electrolyte solution: 0.1 M PBS (pH = 7.0); scan rate: 0.1 V/s.

In order to further explore the reasons why PNIPAM/MWCNTs/GCE detects the temperature-response switch property of DA, the temperature-sensitive behavior of MWCNTs (COOH) was investigated and the MWCNTs (COOH)/GCE were prepared. It can be seen from Figure 8 that the oxidation-peak current and reduction peak do not change significantly with changes in temperature, which further indicates that the temperature-response behavior of PNIPAM/MWCNTs (COOH)/GCE depends on the presence of PNIPAM in the composite film. The temperature-switching mechanism can be explained as follows: PNIPAM is a polymer with temperature-switching effects. When the solution temperature is low, the polymer chain is in a stretched state, which prevents DA in the solution from conducting electron transfer on the electrode surface; when the temperature is high, the polymer segment structure shrinks into clusters, MWCNTs (COOH) give full play to the conductive role, improving the electron-transfer rate, and the electrode is in the “ON” state.

### 3.7. Determination of DA on PNIPAM/MWCNTs(COOH)/GCE

**Figure 9:**
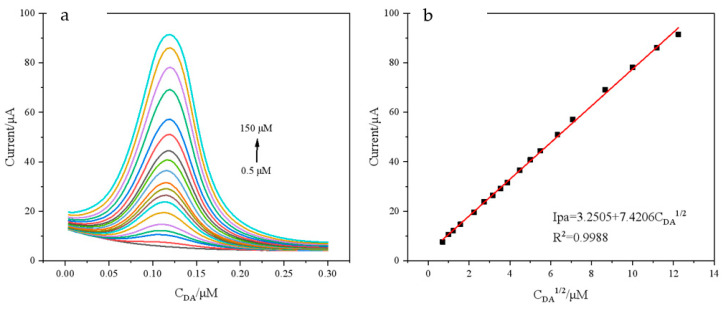
(**a**) DPVs of different concentrations of DA on composite modified electrode and (**b**) linear relationship between oxidation-peak current and corresponding DA concentration. The concentration of DA is 0.5–150 μM, electrolyte solution: 0.1 M PBS (pH = 7.0), scan rate: 0.1 V/s, solution temperature: 40 °C.

DPV was used to investigate the relationship between DA oxidation-peak currents and concentration at a solution temperature of 40 °C. As can be seen in Figure 9a, when DA concentration increases, the peak current also changes. The oxidation-peak current of DA rose linearly with DA concentration in the range of 0.5–150 μM (Figure 9b), and the linear equation is:

Ipa = 3.2505 + 7.4206 C_DA_^1/2^ (μM)(R^2^ = 0.9988)

The detection limit is 0.193 μM. Compared with previously reported detection methods (Table 1), these methods proved that the modified electrode has a good linear range and detection limit, which can better enable the detection of DA.

### 3.8. Stability, Reproducibility and Anti-Interference Test

**Figure 10:**
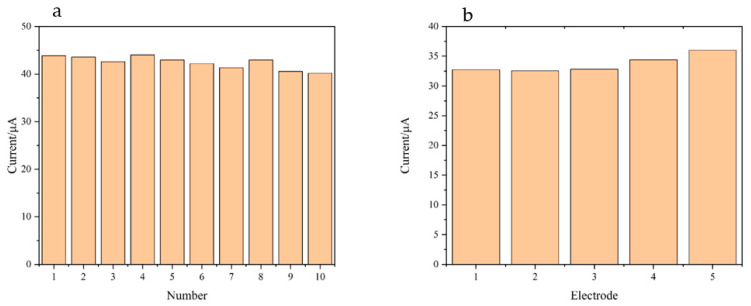
(**a**) PNIPAM/MWCNTs (COOH)/GCE was used for 10 equilibrium experiments; (**b**) DPV responses of five independent electrodes prepared under the same conditions—electrolyte solution: 0.1 M PBS (pH = 7.0), scan rate: 0.1 V/s, solution temperature: 40 °C.

As can be seen in Figure 10a, under the same experimental conditions, the same PNIPAM/MWCNTs (COOH)/GCE-modified electrode was used for 10 equilibrium experiments (DA concentration: 30 μM). It was found that the current value was not significantly weakened and the relative standard deviation (RSD) was 2.88%. This shows that the electrode has good repeatability. Under the same experimental conditions, five different PNIPAM/MWCNTs (COOH)/GCE-modified electrodes were used to detect DA (DA concentration: 20 μM) and the RSD was only 4.43. This shows that the reproducibility is good (Figure 10b).

**Figure 11:**
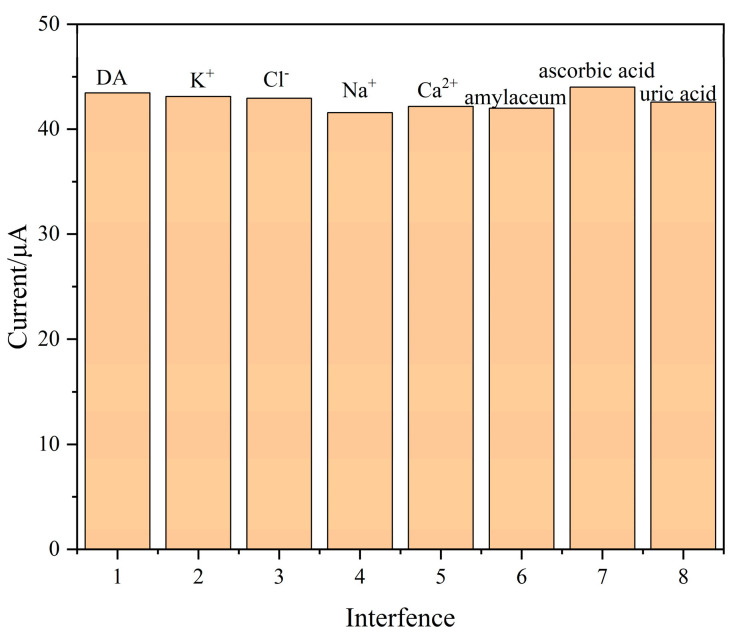
Anti-interference test; electrolyte solution: 0.1 M PBS (pH = 7.0), scan rate: 0.1 V/s, solution temperature: 40 °C.

DPV was used to discuss the influence of interfering substances on DA detection. We added DA to 0.1M PBS solution (DA concentration: 30 μM), before adding more than 5 times’ worth of other substances, including K^+^, Cl^−^, Na^+^, Ca^2+^, Glucose, ascorbic acid and uric acid. As shown in Figure 11, the oxidation-peak current of these substances did not change significantly, indicating that the composite modified electrode has good selectivity for DA.

### 3.9. Actual Sample Detection

In order to explore whether the composite modified electrode can be applied to the detection of actual samples, the recovery rate of DA in serum was calculated using a standard addition method. The experiment found that the recovery rate was between 98.7 and 104.4 (Table 2), indicating that the composite modified electrode has clear application ability.

## 4. Conclusions

In this paper, a temperature-reversible switching electrochemical sensor based on the thermosensitive polymer PNIPAM and MWCNTs (COOH) was constructed for the detection of dopamine. The experimental results show that the sensor can trigger the DA temperature-switch control, which is very sensitive and completely reversible. In addition, the sensor has high stability and a good detection performance for DA and obtained good detection and recovery results in human serum samples. This new switch-type sensor also provides new avenues for the application of thermosensitive polymers. Due to the poor conductivity of polymers, we will focus on developing polymers with better conductivity and heat sensitivity and establish ultra-sensitive and ultra-low LODs for temperature-controlled electrochemical sensors.

## Figures and Tables

**Table 1:** Comparison of different detection methods for DA.

Electrode	Technique Linear Range of DA (μM)	Linear Range of DA (μM)	LOD of HQ (μM)	Ref
PtNCs-MWCNTs-GNPs/GCE	DPV	2–50	0.5	[26]
Pt-MWCNTs/SPE	DPV	0.005–1	0.002	[27]
CNTs/CFE	DPV	5–120.6	0.03	[28]
PNIPAm-GO/GC	DPV	3.9–174	1.3	[29]
PNIPAM/MWCNTs(COOH)/GCE	DPV	0.5–150	0.119	This work

**Table 2:** Detection of dopamine in serum.

Sample	Added/μM	Found/μM	Recovery/%
1	25	24.7	98.8
2	50	49.35	98.7
3	60	62.64	104.4

## Data Availability

The data presented in this study are available on request from the corresponding author.
